# Underlying mechanisms of disruptive mood dysregulation disorder in children: A systematic review by means of research domain criteria

**DOI:** 10.1002/jcv2.12060

**Published:** 2022-02-18

**Authors:** Astrid Brænden, Pål Zeiner, Marit Coldevin, Jan Stubberud, Annika Melinder

**Affiliations:** ^1^ Department of Child and Adolescent Psychiatry Oslo University Hospital Oslo Norway; ^2^ Child and Adolescent Mental Health Research Unit, Department of Research and Innovation Oslo University Hospital Oslo Norway; ^3^ Institute of Clinical Medicine University of Oslo Oslo Norway; ^4^ Nic Waals Institute Lovisenberg Diaconal Hospital Oslo Norway; ^5^ Department of Research Lovisenberg Diaconal Hospital Oslo Norway; ^6^ Department of Psychology University of Oslo Oslo Norway

**Keywords:** child, comorbidity, disruptive mood dysregulation disorder (DMDD), irritability, mechanisms, RDoC, youth

## Abstract

**Background:**

A systematic overview of underlying mechanisms in the new disruptive mood dysregulation disorder (DMDD) diagnosis is needed. The Research Domain Criteria (RDoC) represent a system of six domains of human functioning, which aims to structure the understanding of the nature of mental illnesses. By means of the RDoC framework, the objective of this systematic review is to synthesize available data on children and youths <18 years suffering from DMDD as reported in peer reviewed papers.

**Methods:**

A literature search guided by PRISMA was conducted using Medline, PsychInfo, and Embase, while the RDoC domains were employed to systematize research findings. Risk of bias in the included studies was examined.

**Results:**

We identified 319 studies. After study selection, we included 29 studies. Twenty‐one of these had findings relating to >1 RDoC domain. The risk of bias assessment shows limitations in the research foundation of current knowledge on mechanisms of DMDD.

**Discussion:**

Reviewing self‐report, behavior and neurocircuit findings by means of RDoC domains, we suggest that DMDD youths have a negative interpretation bias in social processes and valence systems. In occurrence of a negative stimuli interpretation, aberrant cognitive processing may arise. However, current knowledge of DMDD is influenced by lack of sample diversity and open science practices.

**Conclusion:**

We found the six RDoC domains useful in structuring current evidence of the underlying mechanisms of DMDD. Important opportunities for future studies in this field of research are suggested. In clinical practice, this comprehensive summary on DMDD mechanisms can be used in psychoeducation and treatment plans.

**Key points**

- Lack of understanding of the underlying mechanisms of clinical irritability defined as disruptive mood dysregulation disorder (DMDD) in children.
- A systematic review by means of Research Domain Criteria (RDoC) is beneficial in understanding DMDD.
- By means of RDoC, this systematic review indicates that DMDD youths have a negative interpretation bias in social processes and valence systems. In conjunction with a negative interpretation of a stimuli, aberrant processing in cognitive systems may occur.
- This systematic review of underlying mechanisms of DMDD can be helpful in psychoeducation and to develop and advance effective treatment programs.
- Future DMDD research should comply with open science practices and ensure sample diversity in this field of research.

## INTRODUCTION

Disruptive mood dysregulation disorder (DMDD) can be regarded as laying above a certain threshold on an irritability continuum which needs treatment (Vidal‐Ribas et al., 2016). This is also known as clinical irritability. In 2013, DMDD was introduced as a new diagnosis in the DSM‐5 within the depressive disorder section (APA, 2013). DMDD originates from the research syndrome “severe mood dysregulation” (SMD; Leibenluft et al., 2003). DMDD and SMD are characterized by severe, recurrent temper outburst (≥3 per week) and by persistently irritable mood (most of the day in ≥12 months) between the outbursts. Most children with SMD meet DMDD criteria (Deveney et al., 2015; Freeman et al., 2016; Stoddard et al., 2015). In line with previous research (Vidal‐Ribas et al., 2016), it seems reasonable to pool these two diagnoses in the present systematic review.

Children with DMDD have severe functional impairment (Copeland et al., 2013; Uran & Kilic, 2020) and adverse outcomes when compared to their treatment‐seeking peers without DMDD and children with no psychiatric disorder (Copeland et al., 2014). Attention deficit hyperactivity disorder (ADHD) is a frequent cooccurring diagnosis in DMDD (Dickstein et al., 2010; Rich et al., 2007; Stoddard et al., 2016). However, irritable mood is not a criteria or characteristic in ADHD (APA, 2013). Thus, clinical irritability (i.e., DMDD) needs individual understanding to tailor appropriate treatment.

Keeping abreast of scientific evidence is necessary to adapt and develop effective treatments by combining high quality evidence with clinical expertise (Guyatt et al., 2000). Systematic reviews can provide trustworthy overviews of current evidence (Cipriani & Barbui, 2006). Of note, a narrative review of DMDD in a Research Domain Criteria (RDoC) perspective has been published (Meyers et al., 2017). Contrary to narrative reviews, however, systematic reviews employ explicit methodological strategies to identify relevant studies. This decreases the possibility of erroneous interpretations of study findings (Cipriani & Barbui, 2006). Thus, for professionals trying to help children suffering from the relatively new DMDD diagnosis, there is a specific need for a systematic review of underlying mechanisms of the condition it represents.

The RDoC provides a framework to examine underlying mechanisms of mental disorders (NIMH, 2021). Contrary to diagnostic manuals with focus on symptoms, the RDoC framework integrates several levels of information to capture function in biological and cognitive systems that may create psychopathology. In fact, RDoC Units of Analysis include genetic, neurocircuit, behavioral and self‐report assessments (NIMH, 2021). For instance, the unit *behavior* includes measurements by cognitive tasks*,* and *neurocircuit* analysis by neuroimaging techniques.

To organize findings of different scientific disciplines, RDoC is divided into six domains of human functioning: *Social Processes* mediates responses to interpersonal settings such as reception of facial communication; *Cognitive Systems* are responsible for cognitive processes such as attention and cognitive control; *Negative* and *Positive Valence Systems* are responsible for responses to aversive and positive motivational situations, respectively; *Arousal and Regulatory Systems* are responsible for context dependent activation of neural systems and homeostatic regulation; and *Sensorimotor Systems*, added recently as a sixth domain, is responsible for motor action and habit.

By means of the RDoC domains and units of analysis, our systematic review synthesizes available data on DMDD, as reported in peer‐reviewed journals and consider bias that may influence the aggregated knowledge on mechanisms of DMDD. While discussing our results, we explore if the RDoC domains provide a comprehensive framework of underlying mechanisms of the condition.

## METHOD

The present review was guided by the Preferred Reporting Items for Systematic Reviews and Meta‐Analyses (PRISMA) for systematic reviews (Moher et al., 2009; Shamseer et al., 2015). The PICO method was used to define eligibility criteria, with the applied formulation: *P*opulation (children <12 years), Indicator (SMD, DMDD, or oppositional defiant disorder with chronic irritability and anger (ODD‐IA; see Supporting Information A), i.e., the equivalent to DMDD in ICD‐11), Comparison group (children without SMD, DMDD, or ODD‐IA), and Outcome (genes, molecules, cells, circuits, physiology, behaviors, reports, and paradigms units of analysis by means of RDoC domains). Due to no involvement of patients or members of the public, an approval from the regional ethical committee was not applied for.

### Search strategy

A systematic literature search by a novel optimization technique when searching for relevant references for systematic reviews (Bramer et al., 2017) was conducted in September 2020 in Embase Classic + Embase, MEDLINE ALL, and APA PsycInfo using Ovid interface. Selection of search terms were guided by our PICO formulation, the diagnostic definitions of children with chronic irritability and frequent temper outburst in NIMH research (SMD; Leibenluft et al., 2003), DSM‐5 (DMDD; APA, 2013), and ICD‐11 (ODD‐IA; Evans, et al., 2017), the RDoC Matrix (NIMH, 2021), and the Schema for determining the optimal order of elements (Bramer et al., 2017).

The search terms were developed and optimized in Embase (see Supporting Information B) and then translated, tested, and reiterated in MEDLINE and PsycInfo according to Bramer et al. (2017). This process was guided by a librarian. The final search terms used in Embase were: ((Disruptive OR severe) ADJ mood dysregulation) OR ((persistent* OR chronic) ADJ3 irritab*) AND (temper ADJ3 (outburst* OR tantrum*)) AND (child* OR youth* OR pediatric*).tw. AND ((cogniti* OR social OR arousal OR threat OR reward OR motor).tw. OR exp cognition/OR exp Social behavior/), (see Supporting Information C for the translation into MEDLINE and PsycInfo).

### Selection criteria

The age criteria were adjusted to <18 years as an initial inspection indicated that only a few numbers of published reports had age criteria as <12 years. Therefore, in this review “youths” refer to children up to 18 years. Exclusion criteria included samples with any individual's age >18 years, full‐text not available, non‐English language, publication type ≠ peer‐reviewed journal article, studies not using DSM or ICD diagnostic criteria, results not related to SMD, DMDD or ODD‐IA or RDoC domains, and if IQ of participants <70. Inclusion and exclusion criteria are detailed in Supporting Information D.

### Identification, selection, and data extraction

Abstracts and titles of the retrieved references were screened independently by the first author. Relevant articles based on selection criteria were screened in full‐text versions, including supplementary online content (see Supporting Information), and the data extraction was done in collaboration with last author (see Supporting Information E, for articles excluded). Identification of the associated RDoC domain(s) with each study was performed based on information about the tasks or methods including outcome measures. For example, studies using a face‐emotion processing task were identified as associated with Social Processes and Valence Systems due to involving both the ability to recognize face emotions and that the identification of an angry face can elicit responses to aversive situations.

### Risk of bias

A practical guide for addressing risk of bias in observational, etiology studies (Savitz et al., 2019) was conducted on the extracted papers. Contrary to more objective tools for assessing intervention studies, which were also considered (see Supporting Information F), this nonalgorithmic, tailored approach emphasizes identifying a few of the most likely influential sources of bias which can provide meaningful insights to consider in the design of new studies. Based on the authors familiarity with the literature, the sample characteristics (e.g., sample sizes and affiliations) of the included studies were hypothesized as having major influence on the overall knowledge on underlying mechanisms of DMDD.

## RESULTS

**FIGURE 1:**
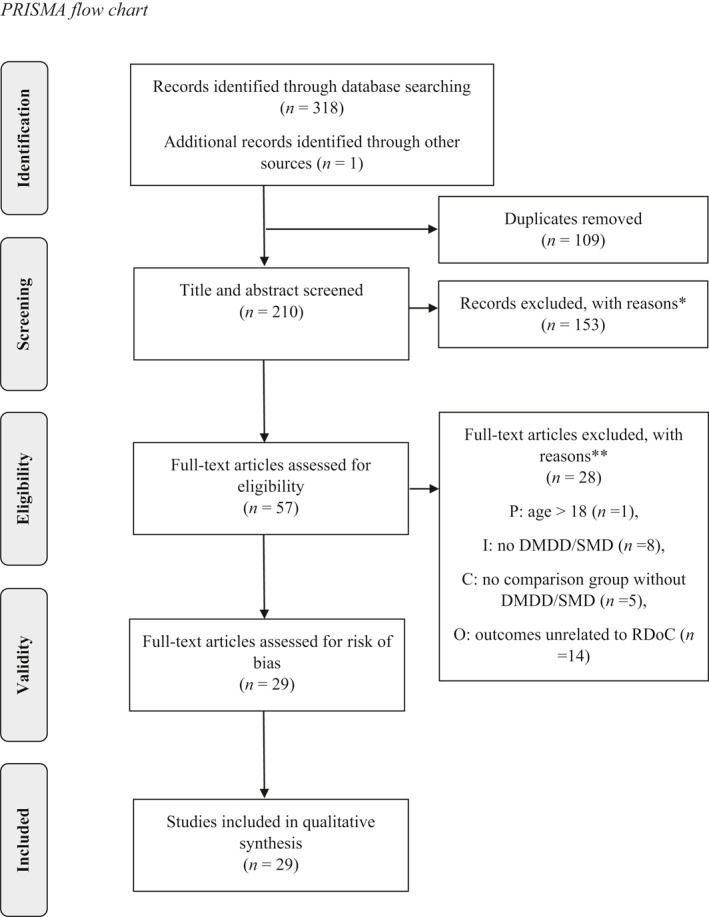
PRISMA flow chart. *Excluded in line with exclusion criteria and PICO formulation. **Reasons elaborated in Supporting Information E

In the following section we present the results, structured by the six RDoC domains. The initial search yielded 318 articles. After duplicates were removed, 209 articles were left for consideration. Manual editing and selection were performed and resulted in 29 included studies. These were assessed for risk of bias. PRISMA flow chart of identification, screening, eligibility assessment and inclusion are presented in Figure 1.

Citations with identified study characteristics are presented in Table 1. For transparency and verifiability purposes, the indicator (DMDD or SMD) used in each study is specified. No study of ODD‐IA was found. The aggregated comorbidity rates of the included studies are 80% for ADHD, 65% for ODD/conduct disorders, 46% for anxiety disorders, and 20% for depressive disorders.

**TABLE 1:** Selected study characteristics

Author	Year	U.S. NIMH affiliated sample?	Age	IQ sample mean	Diagnostic tool	*N* Participants (mean age) % Girls	Objective	Measurements	Result	RDoC domain(s)
Tseng et al. (Including Supporting Information)	2019	Yes	8‐18	112	K‐SADS‐PL	52 DMDD/ARI 42 ANX/ARI40 ADHD/ARI 61 HV/ARI (12.9) 49.7%	Examine associations between irritability and neural activation during responses to frustrating stimuli.	Cued‐attention task differentiating neural activity (whole‐brain fMRI activation and connectivity) in response to (induced) frustration (affective Poser task).	**Behavior:** No reported findings by means of DMDD on attention orienting.	Cognitive systems (attention)
									**fMRI:** Activation: no association with DMDD during attention orienting following frustration. Connectivity (post‐hoc): Higher irritability related to right amygdala‐left culmen functional connectivity in DMDD only (*r* = −0.34, *p* = .02).	Negative valence systems (frustrative Nonreward)
Kircanski et al.	2018	Yes	8‐18	112		54 DMDD/ARI 50 ANX/ARI 37 ADHD/ARI 56 HC/ARI (13.06) 46.2%	Decompose variance of pediatric irritability and anxiety symptoms and to determine neural correlates of these phenothypes during threat orienting.	**Paradigm + fMRI:** Whole‐brain fMRI dot‐probe task assessing attention orienting to angry (i.e., threat) versus neutral faces.	**Behavior:** No behavior differences by diagnosis during threat orienting.	Social processes (reception of facial communication)
									**fMRI:** No distinctive correlates by diagnosis.	Cognitive systems (attention orienting)
										Negative valence systems (threat)
Pagliaccio et al.	2017	Yes	8‐18	113	K‐SADS‐PL	31 DMDD (13.74) 48.4% 25 ADHD 40% 27 HV 56%	Characterize neural and behavioral similarities/differences in attention.	**Paradigm + fMRI:** A fMRI selective attention task, including measurement of accuracy, RT, and ISVRT.	**Behavior:** DMDD and ADHD: Increased RT variability compared with HV when including children excluded from the imaging analysis, but not when analysing the children completing the scan successfully only.	Cognitive systems (attention)
									**fMRI:** DMDD: Specific alterations between RT and right paracentral lobule, superior parietal lobule, fusiform gyrus, and cerebellar culmen. DMDD and ADHD: HV mobilize parietal and related brain regions on long RT trials, DMDD and ADHD fail to deploy this compensatory increase.	
Stoddard et al. (Including Supporting Information)	2017	Yes	8‐17	110	K‐SADS‐PL	37 DMDD 32 ANX 24 ADHD 22 HV (13.2) 44.3%	Determine shared and unique effects of irritability and anxiety on neural responses to facial emotions during fMRI.	**Behavior + fMRI:** fMRI (amygdala connectivity) during gender labeling of varying intensities of face emotions (angry, happy, or fearful).	**Behavior:** No findings reported for diagnostic categories.	Social processes (reception of facial communication)
									**fMRI:** Did not detect associations between irritability‐associated neural responses (i.e., amygdala response or connectivity) and DSM diagnosis or controls.	
Freeman et al.	2016	No	12‐18		KSADS	185 DMDD (10.4) 35% 412 non‐DMDD in a community health sample (10.66) 41%	Examine emotion and behavior problems.	**Self‐report:** Youth self‐report form.	DMDD youths reported more severe aggressive behavior (Cohen's d = 0.61), rule breaking (Cohen's d = 0.42), thought problems (Cohen's d = 0.41), social problems (Cohen's d = 0.31), and attention problems (Cohen's d = 0.27) than youths without DMDD, F(11, 287) = 3.22, p < .001.	Social processes
										Cognitive systems (attention)
Stoddard et al. (Experiment 1)	2016	Yes	8‐18	111	K‐SADS	63 DMDD (13.4) 41.3% 26 HV (13.9) 53.8%	Test whether DMDD have a biased tendency to judge ambiguous facial expression as angry.	**Paradigm:** Interpretation bias task (happy or angry judgements on a continuum).	DMDD required less angry affect in morphs to switch their judgments from predominantly happy to predominantly angry. However, the difference appeared to be driven by an angry judgment bias in DMDD to one male face identity, but not the three others.	Social processes (reception of facial communication)
										Negative (potential threat) and positive (reward valuation) valence systems
Tseng et al.	2016	Yes	8‐18	111	K‐SADS‐PL	17 SMD (14.08) 23.5% 20 HV (14.95) 50% (Different sample than Thomas et al., 2014)	Replicate Thomas et al. (2014) on neural correlates of face emotion processing.	**Paradigm + fMRI:** Affective priming paradigm (masked or unmasked faces: Angry, happy, neutral, or blank oval) and fMRI.	**Behavior:** No differences between SMD and HV in aware or non‐aware face emotion processing.	Social processes (reception of facial communication)
									**fMRI:** SMD increased activation in the parahippocampal gyrus (PHG) and superior temporal gyrus compared to HV when processing angry faces; SMD decreased activation in the insula, PHG, and thalamus compared with HV when processing happy faces. No between‐group differences in amygdala activation.	Negative (potential threat) and positive (reward valuation) valence systems
Uran & Kilic	2015	No	7‐18		K‐SADS‐PL (Turkish version) with SMD module	24 SMD (12.6) 54%67 ADHD‐C (12.1) 31%21 HC (11.7) 67%	Investigate neuropsychological test performance	**Neuropsychological tests**: WCST (executive function), ST‐TBAG (selective attention and response inhibition), TMT (visual attention and task switching), COWAT (verbal fluency and reasoning), CNT (e.g., produce words and cognitive flexibility).	ADHD‐C performances in WCST, TMT, ST‐TBAG and COWAT significantly poorer than control group. SMD performances descriptively intermediate between ADHD‐C and controls.	Cognitive systems (cognitive control)
Perlman et al.	2015	No	6‐9	101	K‐SADS‐PL with SMD module	26 SMD 28 HC	Examine brain activation during reward and frustration.	**Paradigm:** Frustrative emotion task for children including block feedback and emotion rating during fMRI.	**Behavior + self‐report:** RT or self‐reported mood did not differ between SMD and HC. SMD rated themselves as marginally more positive than HC on winning blocks.	Negative (frustrative nonreward) and positive valence (reward Responsiveness) systems
									**fMRI:** SMD more activation in anterior cingulate and middle frontal gyrus during reward and less during frustration (losing condition) compared to HC. Opposite pattern in posterior cingulate. ROI: No effects for the amygdala region.	
Hommer et al.	2014	Yes	7‐17	110	K‐SADS‐PL with SMD module	74 SMD (13.1) 33.8% 42 HC (13.8) 47.6%	Examine attention bias toward threat and happy face stimuli	**Paradigm:** Visual probe paradigm assessing attention bias to emotional faces (face images in pairs: Neutral + a happy, neutral or angry (threatening) face).	SMD bias toward angry faces. No differences in bias toward or away from happy faces.	Social processes (reception of facial communication)
										Negative (potential threat) and positive (reward valuation) valence systems
Thomas et al.	2014	Yes	8‐18	105	K‐SADS‐PL	18 SMD (14.42) 33% 20 BD (15.12) 60% 22 HV (14.75) 59%	Examine aware versus non‐aware face‐emotion labelling deficits.	**Behavior + fMRI + Self‐report:** Affective priming task: fMRI during “Aware” and “non‐aware” priming of shapes by emotional faces (angry, fearful, happy, neutral, blank oval). Subjects rated how much they liked the shape.	**Behavior + Self‐report:** No significant differences between diagnosis on effect of emotions on ratings. BD responded more quickly than SMD and HV.	Social processes (reception of facial communication)
									**fMRI:** All groups show unique activation patterns to different emotions. SMD: Increased activation in non‐aware versus aware conditions in R and L occipital gyrus clusters (vs. Healthy only in R), SMD more activation than HV during viewing angry faces in posterior cingulate, superior temporal gyrus, and middle occipital gyrus, and more activation than BD in the latter. No significant findings in amygdala.	
Thomas et al.	2013	Yes^a^	7‐17	106	K‐SADS‐PL with SMD module	19 SMD (13.42) 47.4% 19 BD (14.22) 47.4% 15 HV (14.98) 66.7%	Compare neural activation during an implicit face‐emotion processing task.	**Behavior + fMRI:** Accuracy of gender identification and RT in facial emotions (neutral, fearful or angry) during fMRI.	**Behavior:** No main effect of diagnosis on accuracy or RT. Observable, but not statistically, differences in accuracy with lowest accuracy in SMD youths (see Table 3 in paper).	Social processes (reception of facial communication)
									**fMRI:** ROI: ↑activity across all expressions in R amygdala. Whole‐brain**:** Deactivation in posterior cingulate cortex, posterior insula, and inferior parietal lobe in response to fearful expressions.	Negative (potential threat) and positive (reward valuation) valence systems
Kim et al.	2013	Yes	8‐18	109	K‐SADS‐PL with SMD module	28 SMD (13.7) 32.1% 22 BD (15.42) 40.9% 22 HC (14.14) 45%	Examine attention to eye regions during facial emotion processing.	**Behavior:** Gaze fixation patterns during a facial emotion labelling task (anger, fear, sadness, happiness, neutral; different emotional levels).	PBD and SMD more labelling errors than HC. SMD did not differ significantly from HC in eye gaze fixation but tended to fall between PBD (which spent less time looking at eyes and made fewer eyes fixation) and HC. ↓fixation to eyes did not correlate with labelling accuracy in SMD.	Social processes (reception of facial communication)
										Negative (potential threat) and positive (reward valuation) valence systems
Deveney et al.	2013	Yes	8‐17	107	K‐SADS‐PL with SMD module	19 SMD (13.6) 21.1% 23 HC (14.3) 52.2%	Examine neural responses to frustration.	**Behavior + Self‐report + fMRI:** Emotional responses, behavior, and ROI fMRI during a cued‐attention task completed under nonfrustrating and frustrating conditions.	**Behavior + Self‐report:** SMD and HC reported ↑frustration and exhibited ↓ ability to shift spatial attention during the frustration condition relative to the nonfrustration condition. These effects of frustration were more marked in SMD, but no other behavior differences between groups. No findings emerged from the arousal ratings. All participants felt more unhappy during the frustration condition than nonfrustrating conditions, with no main effects of groups.	Cognitive systems
									**fMRI:** SMD (compared to HC) had left amygdala hypoactivation and decreased striatal response during negative feedback (vs. positive feedback trials) associated with spatial attention, reward processing, and emotional salience. Did not observe group differences in prefrontal regions.	Negative valence systems (frustrative Nonreward) Arousal/Regulatory systems (arousal)
Thomas et al.	2012	Yes	8‐18	107	K‐SADS‐PL with SMD module	15 SMD (14.53) 27% 19 BD (15.33) 63% 23 HV (14.92) 48%	Compare ability to process subtle changes in facial emotion.	**Behavior + MRI:** Nose width and hostility ratings of facial affect (neutral‐to‐angry and neutral‐to‐happy) during MRI.	**Behavior:** No group (diagnosis) x behavior interactions.	Social processes (reception of facial communication)
									**fMRI:** ROI: Dysfunction in L amygdala activity related to anger on the face (HV show a positive association between L amygdala activity and anger on the face, which SMD or BD does not).	Negative (potential threat) and positive (reward valuation) valence systems
									Whole‐brain: Neutral‐to‐happy: BD and SMD modulated parietal, temporal, and medial‐frontal areas differently from each other and from that in HVs; with ↑facial happiness, SMD patients demonstrated ↑activity.	
Deveney et al.	2012a	Yes	8‐17	108	K‐SADS‐PL with SMD module	67 SMD (12.47) 29.8% 76 BD (12.84) 38.2% 57 HC (13.2) 47.4%	Examine processing of emotional prosody.	**Behavior:** Identify emotional prosody of spoken sentence with neutral content/diagnostic analysis of nonverbal accuracy.	SMD performed more poorly than HC. No interactions group × emotion.	Social processes (reception of facial communication)
Deveney et al.	2012b	Yes	8‐18	112	K‐SADS‐PL with SMD module	26 SMD (13.4) 30.8% 32 BD (14.5) 53.1% 21 HC (13.8) 38.1%	Compare neural activation during motor inhibition task.	**Behavior + fMRI:** ROI based fMRI during stop signal (motor inhibition) task.	**Behavior:** No group differences in motor inhibition.	Cognitive systems (cognitive control; response selection)
									**fMRI:** No difference SMD and HC in any region (the bilateral putamen, caudate, nucleus accumbens, ACC, VLPFC) on neural activation measures.	Sensorimotor systems (motor actions)
Adleman et al.	2012	Yes^a^		110	Leibenluft et al. (2003) SMD criteria and DSM‐IV criteria for BD	78 SMD (12.7) 32.1%/31^b^ SMD 55 BD (14.2) 45.5%/34^b^ BD 68 HV (13.9) 47.1%/27^b^ HV	Compare brain structure development.	**VBM + MRI:** Modulated VBM and structural MRI. Rescan after 2 years.	BD and SMD associated with structural abnormalities in frontal cortex, insula (associated with use of somatic markers in decision‐making and risk‐taking behavior), and basal ganglia. Decreased pre‐SMA volume only in SMD. Concludes that SMD (in contrast to BD) displayed relatively normal structural development.	Cognitive systems (cognitive control, working memory)
										Negative valence systems (loss)
										Positive valence systems (reward learning)
										Sensorimotor systems (motor actions)
Adleman et al.	2011	Yes		110	K‐SADS‐PL with SMD module	22 SMD (13.25) 22.7% 26 BD (14.24) 46.2% 34 HV (14.17) 50%	Examine neural correlates of cognitive flexibility.	**Behavior + fMRI:** Reversal learning task (probabilistic response reversal) assessing cognitive flexibility during ROI (caudate, cingulate gyrus, IFG, ventromedial PFC) based fMRI.	**Behavior:** SMD made more errors across all tasks indicating cognitive flexibility difficulties; their deficit was not limited to the reversal phase.	Cognitive systems (cognitive control, working memory)
									**fMRI:** Hypoactivation in the caudate in SMD and BD, and in the IFG in SMD (not BD), during incorrect responses. Other regions also differentiated SMD from BD: superior parietal lobule and inferior temporal gyrus.	
Rich et al.	2011	Yes^a^	8‐17	108	K‐SADS‐PL	20 SMD (14.15) 25% 20 BD (14.92) 55% 20 controls (14.72) 55%	Examine neural activity to negative affect.	**Paradigm + fMRI + Self‐report:** Affective Posner paradigm (negative vs. positive feedback in blocked goal attainment) during fMRI. RT, response accuracy, self‐reported affect.	**Behavior + Self‐report:** Nonsignificant group × feedback interaction for RT and accuracy: SMD slower RT on negative and positive feedback and worse accuracy on negative feedback than controls but not BD, and SMD worst accuracy on positive feedback compared to both groups. SMD reported greater arousal following negative feedback than BD and controls. SMD and BD reported being less happy than control during the rigged condition.	Negative and positive valence (reward responsiveness) systems
									**fMRI:** SMD responded to negative feedback with significantly greater activation of the anterior cingulate cortex (ACC) and medial frontal gyrus (MFG) than controls and BD.	Arousal/regulatory systems (arousal)
Brotman et al.	2010	Yes	8‐17	109	K‐SADS‐PL	29 SMD (12.94) 41% 37 HC (13.73) 43% 43 PBD (14.81) 60% 18 ADHD (13.87) 28%	Examine amygdala activation during emotion processing.	**Behavior + fMRI + Self‐report:** Passive viewing and rating of emotional and nonemotional aspects of adult faces (happy, angry, fearful, neutral) during (whole‐brain) fMRI. RT.	**Behavior/report:** SMD and PBD by self‐report more afraid of neutral faces than HC. Groups did not differ in hostility or nose‐width ratings. SMD not slower RT in ratings of hostility of neutral faces than HC.	Social processes (reception of facial communication)
									**fMRI:** SMD showed hypoactivation in L amygdala while rating subjective fear of neutral faces, whereas ADHD showed hyperactivation. PBD did not differ from HC. No significant differences in R amygdala between groups. No significant between‐group differences in L or R amygdala during hostility and nose‐width rating contrast.	Negative (potential threat) and positive (reward valuation) valence systems
Dickstein et al.	2010	Yes^a^	7‐17	108	K‐SADS‐PL with SMD module	35 SMD (12.93) 26% 35 NP‐BD (12.6) 34% 42 ANX (12.64) 53% 18 MDD (13.85) 56% 35 HC (13.32) 37%	Determine reversal learning impairments.	**Behavior:** Probabilistic response reversal task.	Impaired reversal learning was present in BD youths, with a trend in those with MDD versus HC. SMD subjects were not significantly different than HC but given the associated effect size (*p* = .13, Cohen's *d* = 0.74), this might represent a type II error. ANX youths did not have reversal learning deficits.	Cognitive systems
Rich et al.	2010	Yes		108	K‐SADS‐PL with SMD module	41 SMD (12.55) 36.6% 57 BD (14.43) 43.9% 33 HC (13.72) 48.48%	Examine impact of emotional stimuli on attention	**Behavior:** Emotional Interruption task: Assesses impact of emotional stimuli (i.e., pictures of varying emotional valence like puppies, a fork, or a shark) on attention. RT and accuracy.	SMD significant reduced attention interference from emotional distracters.	Cognitive systems (attention)
									Comment: SMD youths with greater blunted response to emotional stimuli had greater social dysfunction (parent report and clinician‐assessed).	Negative (potential threat) and positive (reward valuation) valence systems
Rau et al.	2008	Yes		109	K‐SADS‐PL with SMD module	37 SMD (12.5) 37.8% 23 PBD (14.2) 34.8% 31 HC (13.5) 45.2%	Examine decision‐making in reward and punishment processing.	**Behavior:** Decision‐making task in which the participants were asked to choose between two images associated with different levels of reward or punishment.	No between‐group differences in reward and punishment processing.	Negative (potential threat) and positive (reward valuation) valence systems
Rich et al.	2008	Yes	7‐17	109	K‐SADS‐PL with SMD module	31 SMD (12.29) 38.7% 39 NP‐BD (13.99) 46.2% 36 HC (14.34) 54.4%	Examine face‐emotion labelling.	**Paradigm + Behavior:** Emotional expression multimorph task (gradations from neutrality to emotion: Happiness, surprise, fear, sadness, anger, disgust) and behavior (accuracy).	SMD and NP‐BD required significantly more morphs than HV to label correctly disgusted, surprised, fearful, and happy faces. Main effect of covariates (e.g., age) was nonsignificant.	Social processes (reception of facial communication)
										Negative (potential threat) and positive (reward valuation) valence systems
Guyer et al.	2007	Yes	7‐18	106	K‐SADS‐PL with SMD module	39 SMD (11.8) 28.2% 42 BD (12.8) 47.6% 44 ANX and/or MDD (13.1) 47.7% 35 ADHD and/or CD (14.8) 28.6% 92 HC (14.4) 48.9%	Examine face‐emotion labelling.	**Paradigm:** Facial expression recognition subtest from diagnostic analysis of nonverbal accuracy.	SMD (and PBD) significantly more errors. All subjects made more errors on angry than fearful, sad, and happy expressions.	Social processes (reception of facial communication)
										Negative (potential threat) and positive (reward valuation) valence systems
Dickstein et al.	2007	Yes	7‐18	109	K‐SADS‐PL with SMD module	44 SMD (12.2) 30% 50 NP‐BD (13.1) 42% 43 HC (13.6) 36%	Examine cognitive flexibility.	**Behavior:** ID/ED task (i.e., simple and compound reversal) and change task (i.e., inhibiting a prepotent response and substituting a novel response).	SMD and NP‐BD performed worse than controls in compound reversal task. Only NP‐BD difficulties on the simple reversal task.	Cognitive systems (cognitive control, working memory)
Rich et al.	2007	Yes		108	K‐SADS‐PL with SMD module	21 SMD (12.21) 19.05% 35 NP‐BD (12.99) 40% 26 HC (13.74) 87%	Compare behavior and psychophysiological correlates of frustration.	**Behavior + ERP:** Affective Posner task with rigged feedback to induce frustration. Mood response, behavior (RT and accuracy), brain activity (ERP).	**Behavior:** SMD and NP‐BD reported significantly more arousal than HC during frustration. SMD youths had significant lower reaction time during frustration than HC.	Cognitive systems (attention)
									**ERP:** Regardless of emotional context SMD had lower N1 ERP amplitude than HC and NP‐BD, reflecting difficulties in the initial stages of attention. Post hoc analyses demonstrated that this deficit is associated with ODD symptom severity.	Negative valence systems (frustrative nonreward) Arousal/regulatory systems (arousal)
Rich et al.	2005	Yes	7‐17		K‐SADS‐PL with SMD module	19 SMD (11.06) 26.3% 30 NP‐BP (12.7) 33.3% 19 control (13.72) 42.1%	Examine differences in reactivity to reward (monetary) and punishment (aversive noise).	**Paradigm + Behavior + Self‐report:** Lottery startle paradigm. Startle reflex (eyeblink) using electromyographic recordings. Self‐reported mood.	No differences in affect‐modulated startle between controls and SMD or PBD, but SMD and PBD reported greater arousal in the reward condition than controls.	Negative and positive (reward responsiveness) valence systems
										Arousal/regulatory systems (arousal)

Abbreviations: ABC, Aberrant behavior checklist; ACE, Adverse childhood experiences [questionnaire]; ADHD, Attention‐deficit/hyperactivity disorder; ANX, anxiety disorders; ARI, affective reactivity index; ASD, autism specter disorder; (NP or P)BP, narrow‐phenotype or pediatric bipolar disorder; CD: conduct disorder; CGAS, Children's global assessment scale; CNT, category naming test; COWAT, controlled oral word association test; C‐SSRS, The Columbia Suicide Severity Rating Scale; CPRS‐R:L, Conners' Parent Rating Scale‐Revised Long Form; CTRS‐R:L, Conners' Teacher Rating Scale‐Revised Long Form; DMDD, Disruptive Mood Dysregulation Disorder; dlPFC, dorsolateral prefrontal cortex; ERP, Event‐related potentials; FLIS, Family Life Impairment Scale; (f)MRI, (functional) magnetic resonance imaging; HC/HV, healthy controls/comparisons/volunteers; ID/ED, intradimensional/extradimensional shift; IFG, inferior frontal gyrus; ISVRT, intra‐subject variability in RT; K‐SADS‐PL, Schedule for Affective Disorders and Schizophrenia – Present Life Version; MAP‐DB, The Multidimensional Assessment Profile of Disruptive Behavior; MDD, major depressive disorder; (f)MRI, (functional) magnetic resonance imaging; NIH, National Institutes of Health; NIMH, National Institute of Mental Health; OCD, obsessive–compulsive disorder; ODD, oppositional deficit disorder; OFC, orbitofrontal cortex; pre‐SMA, presupplementary motor area; ROI, region of interest; RT, reaction time; SMD, severe mood dysregulation; ST‐TBAG, Stroop test TBAG form; TMT, trail making test; VBM, voxel‐based morphometry; vlPFC, ventrolateral prefrontal cortex; WCST, Wisconsin card sorting test.

^a^ No SMD data published previously.

^b^ Rescanned after 2 years, number of participants.

**FIGURE 2:**
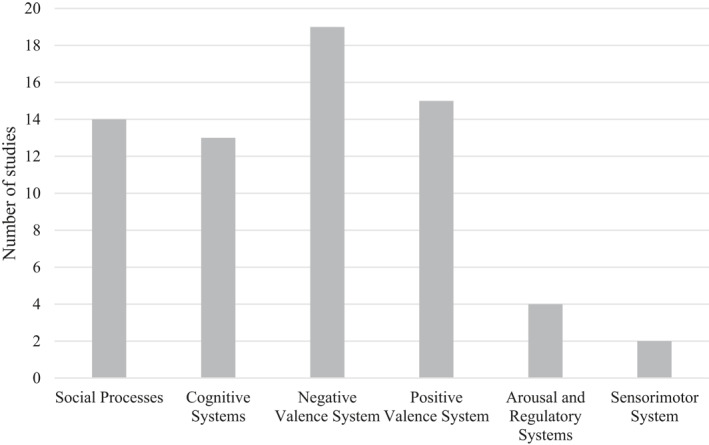
Frequencies of studies by RDoC domains. As the sum of studies are more than the actual number of included studies, several findings have been identified as relevant to more than one RDoC domain

Twenty‐one of the included studies were identified as related to more than one RDoC domain. Number of articles per RDoC domain is presented in Figure 2.

### Risk of bias

As presented in Table 1, the study samples are characterized by a predominantly U.S. NIMH affiliation and insufficient information to identify similar sample sets (see also Supporting Information G). The sample size median of the included studies is 91 participants and the median of participants in each comparison group is 32. Some studies have not corrected for multiple comparisons in their analyses (Guyer et al., 2007; Thomas et al., 2013, 2014) whereas other have not provided adequate information to conclude whether such corrections are done (Adleman et al., 2011; Brotman et al., 2010; Deveney, Brotman, et al., 2012a; Freeman et al., 2016; Kim et al., 2013; Pagliaccio et al., 2017; Rich et al., 2011). The aggregated IQ score of the samples is 109, and 111 for the control groups and 106 for the SMD and DMDD groups. There is a lack of knowledge regarding socioeconomic factors as only five studies provide such characteristics (Kircanski et al., 2018; Perlman et al., 2015; Stoddard et al., 2017; Tseng et al., 2019; Uran & Kilic, 2015). Consequences for current knowledge on DMDD mechanisms are detailed in the discussion.

### Social processes

Fourteen studies are identified as describing Social Processes. DMDD youths rate themselves as having significantly more social problems than youths with other psychiatric disorders (Freeman et al., 2016). Also, SMD youths report being more afraid of neutral faces than controls (Brotman et al., 2010). On behavior level, SMD youths have a bias toward angry faces, but not toward or away from happy faces (Hommer et al., 2014), and they have difficulties in identifying emotional prosody (Deveney, Brotman, et al., 2012a). Three studies find that SMD youths have face‐emotion labelling difficulties (Guyer et al., 2007; Kim et al., 2013; Rich et al., 2008), whereas others do not (Thomas et al., 2012, 2014; Tseng et al., 2016). One study finds observable, but not statistically, differences in implicit face‐emotion accuracy with SMD having the lowest accuracy compared to healthy controls (HC) and youths with BD (Thomas et al., 2013).

On a neurocircuit level of analysis, some studies find abnormalities in left or right amygdala activation during face‐emotion processing (Brotman et al., 2010; Thomas et al., 2012, 2013) whereas others do not (Kircanski et al., 2018; Stoddard et al., 2017; Thomas et al., 2014; Tseng et al., 2016). Noteworthy, differences in face‐emotion processing tasks makes it difficult to compare these results directly. For example, one study examines attention orienting to angry versus neutral faces (Kircanski et al., 2018) whereas another investigates passive viewing and rating of emotional and nonemotional aspects of happy, angry, fearful, and neutral faces (Brotman et al., 2010). Similar ambiguity yields for other neurocircuit findings. For example, some studies do not detect association between neural activity using fMRI and threat orienting (Kircanski et al., 2018) or responses to face‐emotions (Stoddard et al., 2017) in DMDD, whereas others find that SMD youths show hyperactivation in superior temporal gyrus compared to HC when viewing angry faces (Thomas et al., 2014; Tseng et al., 2016).

In sum, social processing difficulties are present in DMDD youths by self‐report. By task performance (e.g., behavior) some studies find face‐emotion labelling deficits in these youths whereas others do not. There is, however, indication of a bias toward threatening or angry faces. On neurocircuit level of analysis, hyperactivation in superior temporal gyrus when viewing angry faces is demonstrated, whereas amygdala activity findings during face‐emotion processing are inconsistent, possibly due to different processing tasks.

### Valence systems

As relevant to Negative Valence System and Positive Valence System, 19 and 15 studies respectively are identified. Responses in negative or positive valence systems can arise from the valuation of angry or happy faces, indicating an overlap between valence systems and social processes. We refer to the social processes section for results associated with face emotion‐processing.

Our results show that SMD youths do not have abnormalities on behavior level by task performance in reward or punishment processing (Rau et al., 2008; Rich et al., 2005). In response to frustration, SMD youths report high levels of arousal (Rich et al., 2007, 2011) whereas another study finds no differences in mood ratings in response to reward or frustration (Perlman et al., 2015). However, one study does not report arousal ratings using the same task paradigm in DMDD youths as Rich and colleagues (Tseng et al., 2019). There is some indication of SMD youths having slower reaction time to frustration, but the results are equivocal (Rich et al., 2007, 2011). In response to frustration one study finds increased neuronal activity in anterior cingulate cortex (ACC) and medio‐frontal gyrus (MFG) in SMD youths using magnetoencephalography (Rich et al., 2011), whereas another study finds no neuronal activity or connectivity abnormalities in DMDD using fMRI (Tseng et al., 2019). Furthermore, a third study finds that SMD have decreased activation during frustration in ACC and MFG and increased activation during reward (Perlman et al., 2015). The same study found no effects in the amygdala.

One study finds that SMD youths have reduced attention interference from (nonsocial) emotional distracters (Rich et al., 2010). However, during frustration SMD youths demonstrates a disability in shifting spatial attention with associated left amygdala hypoactivation and decreased striatal response, but no prefrontal regions abnormalities (Deveney et al., 2013). Furthermore, a recent study finds no abnormalities in attention orienting to, or distraction by, threat in DMDD youths on behavioral or neural level (Kircanski et al., 2018).

Taken together, in DMDD there is indication of abnormal responses to frustration by self‐report, behavior paradigms and neurocircuit activity. However, the results are ambiguous in terms of different results on reports of arousal in response to frustration. No abnormalities in reward or punishment processing are demonstrated on behavior level.

### Cognitive systems

Thirteen studies are identified as relating to Cognitive Systems. By self‐report, one study finds that DMDD youths experience more attentional problems than youths without DMDD in psychiatric clinical assessment (Freeman et al., 2016). Two studies have examined attention without social or emotional interference on behavior level. These studies indicate that DMDD youths might have impairments in selective attention and visual attention, but the results are equivocal (Pagliaccio et al., 2017; Uran & Kilic, 2015). On a neurocircuit level, youths with DMDD or ADHD fail to mobilize parietal and related brain regions during long reaction time in a selective attention task (Pagliaccio et al., 2017). The association between attention and frustration or face‐emotion processing appears ambiguous (Deveney et al., 2013; Hommer et al., 2014; Kim et al., 2013; Kircanski et al., 2018; Rich et al., 2007, 2010; Tseng et al., 2019), and is described under social processes and valence systems as overlap with these domains systems exists. Additionally, on behavior level (performance‐based), SMD youths seem to have impaired cognitive flexibility and normal motor inhibition abilities, but the results are obscure (Adleman et al., 2011; Deveney, Connolly, et al., 2012b; Dickstein et al., 2007, 2010; Uran & Kilic, 2015).

In brief, current research suggests that DMDD youths have impaired cognitive flexibility in addition to attention difficulties.

### Arousal, regulatory, and sensorimotor systems

Few findings (four and two, respectively) associated with Arousal/Regulatory and Sensorimotor Systems were identified. Some studies find that SMD youths report more arousal than HC during frustration (Rich et al., 2007, 2011) whereas others do not (Deveney et al., 2013). During a motor inhibition task, no differences between SMD and HC, is found on behavior or neural activation measures (Deveney, Connolly, et al., 2012b).

## DISCUSSION

By means of RDoC domains and self‐report, behavior and neurocircuit level of analysis, our results indicate that DMDD youths have abnormalities in social processes with a bias toward angry faces, valence systems with aberrant frustration response, and cognitive systems, where impairments in cognitive flexibility were observed. Notably, overlap between these domains are present and the results appear ambiguous. Below we discuss our findings.

Social processing findings, showing that DMDD youths have a bias toward angry faces and difficulties in ascertaining the correct emotional tone of a spoken sentence, specifically suggest abnormal processing in the *Reception of Communication*, a subconstruct of social processes. Evidently, the capacity to convey their feelings (the *Production of Communication*) is unexamined. Some researchers have argued that SMD youths have an extended deficit in labeling emotions of others to include deficits in emotional self‐monitoring (Stoddard, et al., 2014). Hence, DMDD youths might have more general difficulties in processing of self and others. Comparable scores on social awareness in SMD and autism spectrum disorder (Sturm, et al., 2018) highlights this possibility. However, research on DMDD youths understanding of the self and mental states of others is scarce.

Problems with attention and cognitive control (i.e., subconstructs of cognitive systems) is indicated in DMDD youths but the results are equivocal. Social and valence mechanisms, which can provide insights to cognitive processes due to overlap between the domains, point to possible perception bias in reception of communication and frustration inducing stimuli in these youths. A recent study suggests that youths with ADHD Combined Type are more inattentive than DMDD youths, but DMDD youths more emotionally labile than ADHD youths (Uran & Kilic, 2020). These results indicate a difference in mechanisms related to attention with DMDD having a context specific and ADHD having a general deficit in attention. Notably, this also imply that emotional hyperarousal, that is, hyper‐lability, likely linked to the presence or activation of the perception bias, might be a unique mechanism of DMDD. In essence, DMDD might be particularly characterized by emotional hyperarousal, and ADHD by cognitive hyperarousal. Indeed, the same study found that children with ≥2 psychiatric comorbidities in DMDD and ADHD Combined Type had significantly higher scores in indexes on “Oppositional,” “Inattention,” and “ADHD index.” This imply that the inattention symptomology worsens when a general inattention deficit and emotional lability interact making inattention problems higher in both emotional and nonemotional contexts. Thus, it may be feasible to examine attention and constructs such as perception and cognitive control in conjunction with other domains as interaction may create the specific symptomology observed in DMDD.

Previous literature argues that DMDD youths exhibit low frustration tolerance, supporting the role of *frustrative nonreward* processes from the negative valence domain (Meyers et al., 2017). As apparent from the present review, there is not conclusive evidence for abnormal responses to frustration in these youths. By building on previous work (Brotman et al., 2017; Meyers et al., 2017; Stringaris et al., 2018) and accounting for the reasoning in the present systematic review, it is still possible to argue that DMDD youths have a specific negative interpretation bias in both social processes and valence systems (i.e., “hot” cognitive abnormalities), and that “cold” cognitive system abnormalities occur primarily in conjunction with such interpretations. Seemingly inconsistent results of the association between cognitive processes with or without emotional interference (e.g., the involvement of amygdala and the ACC, face‐emotion labelling deficits, and responses to frustration), might depend on the instrument's achievement in eliciting “hot” and “cold” processes. Consistent with a developmental system perspective, DMDD youths might have an immature socioemotional system relative to their cognitive control system, that is, a significant discrepancy in the maturation and connections of their socioemotional system and cognitive control system (Casey et al., 2008; Steinberg, 2008). Findings of suicidal attempt as unplanned and impulsive in DMDD (Benarous, et al., 2020) speaks to the potential severe consequences of an immature socioemotional system on cognitive control.

Despite the fact that hyperarousal was a criterion for SMD, we conclude that research on arousal and regulatory processes is scarce. DSM‐5 did not include hyperarousal as a DMDD criteria, but as sensitivity to stimuli and activation of neural systems is intertwined with social, valence and cognitive processes and perceptions, regulatory processes and arousal are clearly relevant. If emotional (hyper)lability linked to a negative interpretation tendency is a mechanism of DMDD, as we propose, continued efforts and innovative methods are needed to examine this unique chain of interactions and neural sensitivity. As initially suggested by the SMD criteria, hyperarousal might be a key element in clinical irritability and anger.

Researchers have raised fundamental concerns regarding the validity of DMDD as a diagnostic group (Malhi & Bell, 2019). This speaks to the importance of studying unique mechanisms of DMDD to clarify this issue, which is challenging due to the high comorbidity rates. As advocated by the RDoC initiative dimensional approaches to psychopathology are beneficial. Measuring irritability, the Affective Reactivity Index (ARI; Stringaris, et al., 2012) have been used in recent research with this purpose. By such, different neural associations of irritability levels and diagnostic groups are observed (Kircanski et al., 2018; Tseng et al., 2019). Acknowledging DMDD as lying above a certain threshold on an irritability continuum (Vidal‐Ribas et al., 2016), dimensional irritability measurements can both help to understand normative variation and improve our understanding of mechanisms of clinical irritability across diagnostic groups.

By means of RDoC domains, studies examining associations between DMDD and childhood stressors (Basu & Isaacs, 2019; Benarous et al., 2020; Copeland et al., 2013; Rich et al., 2008; Uran & Kilic, 2020) did not get a natural affinity in our systematic review. Childhood maltreatment is associated with altered stress responses and symptoms coinciding with DMDD such as irritability and a negative interpretation bias (Bérubé et al., 2021; Teicher et al., 2003). Even if reports of childhood stressors not directly uncover regulatory and arousal mechanisms in DMDD, such results are important in understanding the potential trajectories from normal to abnormal irritability and anger. In keeping with the RDoC framework, future research may benefit from direct measurement of stress responses, such as cortisol levels, in clinical irritability.

Childhood stressors are linked to socioeconomic factors (Farah, 2017; Johnson et al., 2016). In our risk of bias assessment, we identified a pressing issue to address such factors to understand the etiology of clinical irritability. Interestingly, our bias assessment shows that the U.S. NIMH serves a prevailing role in sample recruitment in current DMDD research and that studies published before 2016 have not provided sufficient information to identify their sample sets. This raises the possibility of several research results being based on the same or part of the same sample, that is, rig mar rolling (Agwor & Adesina, 2017), which in turn resulted in the inclusion of DMDD in DSM‐5, and in the present context; in the sampling of papers for our review. The sample sizes and the number of participants in each comparison group also indicates that type 2 errors may influence the individual and aggregated results. Failure to statistically control against multiple comparisons and lack of transparency regarding statistical methods means that type 1 errors may also be present. Furthermore, the IQ levels makes us question the generalizability of current knowledge on mechanisms of DMDD. Indeed, this does not contradict the importance of assessing the results of this review. Existing research constitutes an advantageous foundation for planning new research and treatment approaches. The sample characteristics assessed in this review convey exiting opportunities for future studies to increase sample diversity from different work groups and geographical areas, include larger sample sizes to increase statistical power, to ensure appropriate statistical methods, and to comply with open science practices ensuring transparency and verifiability. Such efforts will provide significant improvements in our understanding on mechanisms of DMDD.

### Limitations

By focusing on the six RDoC domains, other interesting results might have been insufficiently emphasized as exemplified with childhood stressors. Also, it is difficult to identify appropriate measures of RDoC constructs (Watson et al., 2017). Whether and how units of analysis relate to RDoC domains and constructs is ambiguous. Nevertheless, by tabulating the results in this review including the associated RDoC domains, investigating our evaluations are readily available.

Even though SMD was used to understand DMDD in the present review, the relatively small difference between DMDD and SMD criteria might be significant and becomes evident when examining youths on a group level. Nevertheless, due to scarcity of DMDD research, it seemed reasonable to include SMD research to gain insight into DMDD mechanisms. Indeed, RDoC advocates dimensional approaches to psychopathology. As such, a categorical comparison by means of RDoC can be questionable. However, an overview of underlying mechanisms specific to DMDD as provided in this systematic review is necessary to clinicians giving psychoeducation and treatment to youths currently being diagnosed with DMDD. We argue that both diagnostic categories and dimensional approaches to psychopathology contributes to our understanding mental disorders and serves different and compatible purposes to researchers and clinicians.

Our systematic review does not include non‐English or non‐published studies and the results may be influenced by selection and publication bias. Potential biases influencing the overall knowledge on mechanisms of DMDD might also be present beyond those addressed here. For example, we did not examine whether the measurements of the included studies were validated or reliable, neither the ecological validity, nor weighted the importance of each study result against each other. Nevertheless, this systematic review provides a comprehensive summary of new knowledge on mechanisms of DMDD and important considerations and guidelines for future studies on this topic.

### Conclusion

By building on previous literature and accounting for the results of this systematic review by means of RDoC, we argue that DMDD youths have a negative interpretation bias in social processes and valence systems, that is, primarily abnormalities in emotion (vs. cognitive) related perceptions and underlying processes. This is in line with a developmental system perspective with DMDD youths having an immature socioemotional system relative to their cognitive system, and accounts for RDoC domains as overlapping and highly interconnected. Important areas for future research on DMDD mechanisms are continued examination of “hot” and “cold” processes and interactions, using both diagnostic and dimensional approaches to irritability. Additionally, there is a pressing issue to address the associations with arousal and regulatory systems and childhood stressors. Already, the description of DMDD mechanisms in the present review can be helpful in psychoeducation and to develop and advance effective treatment programs. This includes support for clinical trials targeting the negative interpretation bias to improve irritability and anger tolerance and promoting emotion regulation techniques. Importantly, all future studies on mechanisms of DMDD are encouraged to increase sample diversity and statistical transparency, following open science practices, which can lead to significant improvements in this new field of research.

## CONFLICT OF INTERESTS

The authors have declared that they have no competing or potential conflicts of interest.

## ETHICS CONSIDERATION

Due to no involvement of patients or members of the public, an approval from the regional ethical committee was not applied for.

## AUTHOR CONTRIBUTIONS

**Astrid Brænden:** Conceptualization, Formal analysis, Investigation, Methodology, Validation, Visualization, Writing‐original draft, Writing‐review & editing. **Pål Zeiner:** Conceptualization, Investigation, Methodology, Supervision, Writing‐review & editing. **Marit Coldevin:** Conceptualization, Investigation, Validation, Writing‐review & editing. **Jan Stubberud:** Methodology, Supervision, Validation, Writing‐review & editing. **Annika Melinder:** Conceptualization, Formal analysis, Funding acquisition, Investigation, Methodology, Project administration, Supervision, Validation, Writing‐review & editing.

## Supporting information

Supporting Information 1

## Data Availability

Data sharing not applicable—no new data generated, or the article describes entirely theoretical research.

## References

[jcv212060-bib-0001] Adleman, N. , Kayser, R. , Dickstein, D. , Blair, R. , Pine, D. , & Leibenluft, E. (2011). Neural correlates of reversal learning in severe mood dysregulation and pediatric bipolar disorder. Journal of the American Acadamy of Child and Adolecent Psychiatry, 50(11), 1173–1185. 10.1016/j.jaac.2011.07.011 PMC3206630 22024005

[jcv212060-bib-0002] Agwor, T. , & Adesina, O. (2017). Ethical issues for consideration in conducting research in the social and behavioural sciences. The International Journal of Humanities & Social Studies, 203(12). 10.2015/AJPH.2013.301599

[jcv212060-bib-0003] American Psychiatric Association . (2013). Depressive disorders in diagnostic and statistical manual of mental disorders (5th ed.). 10.1176/appi.books.9780890425596.dsm04

[jcv212060-bib-0004] Basu, S. , & Isaacs, A. (2019). Profile of transcultural patients in a regional Child and Adolescent Mental Health Service in Gippsland, Australia: The need for a multidimensional understanding of complexities. Social Psychiatry, 65(3), 217–224. 10.1177/0020764019835264 30880536

[jcv212060-bib-0005] Benarous, X. , Renaud, J. , Breton, J. , Cohen, D. , Réal, L. , & Guilé, J.‐M. (2020). Are youths with disruptive mood dysregulation disorder different from youths with major depressive disorder or persistent depressive disorder? Journal of Affective Disorders, 265, 207–215. 10.1016/j.jad.2020.01.020 32090743

[jcv212060-bib-0006] Bérubé, A. , Turgeon, J. , Blais, C. , & Fiset, D. (2021), Emotion recognition in adults with a history of childhood maltreatment: A systematic review. Trauma, Violence, & Abuse. 10.1177/15248380211029403 PMC9660286 34238064

[jcv212060-bib-0007] Bramer , W. , de Jonge, G. , Rethlefsen, M. , Mast, F. , & Kleijnen, J. (2017). A systematic approach to searching: An efficient and complete method to develop literature searches. Journal of the Medical Library Association, 106(4). 10.5195/jmla.2018.283 PMC6148622 30271302

[jcv212060-bib-0008] Brotman, M. A. , Kircanski, K. , & Leibenluft, E. (2017). Irritability in children and adolescents. Annual Review of Clinical Psychology, 13, 317–341. 10.1146/annurev-clinpsy-032816-044941 PMC13292837 28482689

[jcv212060-bib-0009] Brotman, M. , Rich, B. , Guyer, A. , Lunsford, J. , Horsey, S. , Reising, M. , Thomas, L. A. , Fromm, S. J. , Towbin, K. , Pine, D. S. , & Leibenluft, E. (2010). Amygdala activation during emotion processing of neutral faces in children with severe mood dysregulation versus ADHD or bipolar disorder. American Journal of Psychiatry, 167(1), 61–69. 10.1176/appi.ajp.2009.09010043 19917597 PMC3075433

[jcv212060-bib-0010] Casey, B. J. , Getz, S. , & Galvan, A. (2008). The adolescent brain. Developmental Review, 28, 62–77. 10.1016/j.dr.2007.08.003 18688292 PMC2500212

[jcv212060-bib-0011] Cipriani, A. , & Barbui, C. (2006). What is a systematic review? Epidemiologia e Psichiatria Sociale, 15(3). 10.1017/S1121189X00004413 17128619

[jcv212060-bib-0012] Copeland, W. , Angold, A. , Costello, J. , & Egger, H. (2013). Prevalence, comorbidity, and correlates of DSM‐5 proposed disruptive mood dysregulation disorder. American Journal of Psychiatry, 170(2), 173–179. 10.1176/appi.ajp.2012.12010132 23377638 PMC3573525

[jcv212060-bib-0013] Copeland, W. , Shanahan, L. , Egger, H. , Angold, A. , & Castello, E. (2014). Adult diagnostic and functional outcomes of DSM‐5 disruptive mood dysregulation disorder. American Journal of Psychiatry, 171(6), 668–674. 10.1176/appi.ajp.2014.13091213 24781389 PMC4106474

[jcv212060-bib-0014] Deveney, C. , Brotman, M. , Decker, A. , Pine, D. , & Leibenluft, E. (2012a). Affective prosody labeling in youths with bipolar disorder or severe mood dysregulation. Journal of Child Psychology and Psychiatry, 53(3). 10.1111/j.1469-7610.2011.02482.x PMC4472432 22029604

[jcv212060-bib-0015] Deveney, C. , Connolly, M. , Haring, C. , Bones, B. , Reynolds, R. , Kim, P. , Pine, D. , & Leibenluft, E. (2013). Neural mechanisms of frustration in chronically irritable children. American Journal of Psychiatry, 170(10), 1186–1194. 10.1176/appi.ajp.2013.12070917 23732841 PMC3938281

[jcv212060-bib-0016] Deveney, C. , Connolly, M. , Jenkins, S. , Kim, P. , Fromm, S. , Pine, D. , & Leibenluft, E. (2012b). Neural recruitment during failed motor inhibition differentiates youths with bipolar disorder and severe mood dysregulation. Biological Psychology, 89(1), 148–155. 10.1016/j.biopsycho.2011.10.003 22008364 PMC3245776

[jcv212060-bib-0017] Deveney, C. M. , Hommer, R. E. , Reeves, E. , Stringaris, A. , Hinton, K. E. , Haring, C. T. , Ribas, P. V. , Towbin, K. , Brotman, M. A. , & Leibenluft, E. (2015). A prospective study of severe irritability in youths: 2‐ and 4‐year follow‐up. Depression and Anxiety, 32(5), 364–372. 10.1002/da.22336 25504765 PMC10530700

[jcv212060-bib-0018] Dickstein, D. , Finger, E. , Brotman, M. , Rich, B. , Pine, D. , Blair, J. , & Leibenluft, E. (2010). Impaired probabilistic reversal learning in youths with mood and anxiety disorders. Psychological Medicine, 40(7). 10.1017/S0033291709991462 PMC3000432 19818204

[jcv212060-bib-0019] Dickstein, D. , Nelson, E. , McClure, E. , Grimley, M. , Knopf, L. , Brotman, M. , Rich, B. A. , Pine, D. S. , & Leibenluft, E. (2007). Cognitive flexibility in phenotypes of pediatric bipolar disorder. Journal of the American Academy of Child & Adolescent Psychiatry, 46(3), 341–355. 10.1097/chi.0b013e31802d0b3d 17314720

[jcv212060-bib-0020] Evans, S. , Burke, J. , Roberts, M. , Fite, P. , Lochman, J. , de la Pena, F. , & Reed, G. (2017). Irritability in child and adolescent psychopathology: An integrative review for ICD‐11. Clinical Psychology Review, 53, 29–45. 10.1016/j.cpr.2017.01.004 28192774

[jcv212060-bib-0021] Farah, M. J. (2017). The neuroscience of socioeconomic status: Correlates, causes, and consequences. Neuron, 96(1), 56–71. 10.1016/j.neuron.2017.08.034 28957676

[jcv212060-bib-0022] Freeman, A. , Youngstrom, E. , Youngstrom, J. , & Findling, R. (2016). Disruptive mood dysregulation disorder in a community mental health clinic: Prevalence, comorbidity and correlates. Journal of Child and Adolescent Psychopharmacology, 26(2), 123–130. 10.1089/cap.2015.0061 26745325 PMC4800380

[jcv212060-bib-0023] Guyatt, G. H. , Haynes, R. B. , Jaeschke, R. Z. , Cook, D. J. , Green, L. , Naylor, D. , Wilson, M. C. , & Richardson, S. (2000). Users' guides to the medical literature: XXV. Evidence‐based medicine: Principles for applying the users' guides to patient care. JAMA. 284(10), 1290. 10.1001/jama.284.10.1290 10979117

[jcv212060-bib-0024] Guyer, A. , McClure, E. , Adler, A. , Brotman, M. , Rich, B. , Kimes, A. , Pine, D. S. , Ernst, M. , & Leibenluft, E. (2007). Specificity of facial expression labeling deficits in childhood psychopathology. Journal of Child Psychology and Psychiatry, 48(9), 863–871. 10.1111/j.1469-7610.2007.01758.x 17714371

[jcv212060-bib-0025] Hommer, R. , Meyers, A. , Stoddard, J. , Connolly, M. , Mogg, K. , Bradley, B. , Brendan, P. , Pine, D. S. , Leibenluft, E. , & Brotman, M. (2014). Attention bias to threat faces in severe mood dysregulation. Depression and Anxiety, 31(7). 10.1002/da.22145 PMC3933451 23798350

[jcv212060-bib-0026] Johnson, S. B. , Riis, J. K. , & Noble, K. G. (2016). State of the art review: Poverty and the developing brain. Pediatrics, 137(4). 10.1542/peds.2015-3075 PMC4811314 26952506

[jcv212060-bib-0027] Kim, P. , Arizpe, J. , Rosen, B. , Razdan, V. , Haring, C. , Jenkins, S. , Deveney, C. M. , Brotman, M. A. , Blair, R. J. , Pine, D. S. , Leibenluft, E. , Baker, C. I. , & Arizpe, J. (2013). Impaired fixation to eyes during facial emotion labelling in children with bipolar disorder or severe mood dysregulation. Journal of Psychiatry & Neuroscience, 38(6), 407–416. 10.1503/jpn.120232 23906351 PMC3819155

[jcv212060-bib-0028] Kircanski, K. , White, L. , Tseng, W.‐L. , Wiggins, J. , Frank, H. , Sequeira, S. , Zhang, S. , Abend, R. , Towbin, K. E. , Stringaris, A. , Pine, D. S. , Leibenluft, E. , & Brotman, M. (2018). A Latent variable approach to differentiating neural mechanisms of irritability and anxiety in youth. JAMA Psychiatry, 75(6), 631. 10.1001/jamapsychiatry.2018.0468 29625429 PMC6137523

[jcv212060-bib-0029] Leibenluft, E. , Charney, D. , Towbin, K. , Bhangoo, R. , & Pine, D. (2003). Defining clinical phenotypes of Juvenile Mania. The Americal Journal of Psychiatry, 160(3), 430–437. 10.1176/appi.ajp.160.3.430 12611821

[jcv212060-bib-0030] Malhi, G. S. , & Bell, E. (2019). Fake views: DMDD, indeed. Australian and New Zealand Journal of Psychiatry, 53(7), 706–710. 10.1177/0004867419863162 31282190

[jcv212060-bib-0031] Meyers, E. , DeSerisy, M. , & Roy, A. (2017). Disruptive mood dysregulation disorder (DMDD): An RDoC perspective. Journal of Affective Disorders, 216, 117–122. 10.1016/j.jad.2016.08.007 27554606 PMC5305694

[jcv212060-bib-0032] Moher, D. , Liberati, A. , Tetzlaff, J. , & Altman, D. (2009). Preferred reporting items for systematic reviews and metaanalyses: The PRISMA statement. BMJ, 339(7716), b2535. 10.1136/bmj.b2535 19622551 PMC2714657

[jcv212060-bib-0033] National Institute of Mental Health . (2021, November 10). About RDoC. https://www.nimh.nih.gov/research/research‐funded‐by‐nimh/rdoc/about‐rdoc

[jcv212060-bib-0034] Pagliaccio, D. , Wiggins, J. , Adleman, N. , Curhan, A. , Zhang, S. , Towbin, K. , Brotman, M. A. , Pine, D. S. , & Leibenluft, E. (2017). Behavioral and neural sustained attention deficits in disruptive mood dysregulation disorder and attention‐deficit/hyperactivity disorder. Journal of the American Academy of Child & Adolescent Psychiatry, 56(5), 426–435. 10.1016/j.jaac.2017.02.008 28433092 PMC5407501

[jcv212060-bib-0035] Perlman, S. B. , Jones, B. M. , Wakschlag, L. S. , Axelson, D. , Birmaher, B. , & Phillips, M. L. (2015). Neural substrates of child irritability in typically developing and psychiatric populations. Developmental cognitive neuroscience, 14, 71–80. 10.1016/j.dcn.2015.07.003 26218424 PMC4536125

[jcv212060-bib-0036] Rau, G. , Blair, K. , Berghorst, L. , Knopf, L. , Skup, M. , Luckenbaugh, D. , Pine, D. A. , Blair, R. J. , & Leibenluft, E. (2008). Processing of differentially valued rewards and punishments in youths with bipolar disorder or severe mood dysregulation. Journal of Child and Adolescent Psychopharmacology, 18(2), 185–196. 10.1089/cap.2007.0053 18439115 PMC2683389

[jcv212060-bib-0037] Rich, B. , Bhangoo, R. , Vinton, D. , Berhorst, L. , Dickstein, D. , Grillon, C. , Davidson, R. J. , & Leibenluft, E. (2005). Using affect‐modulated startle to study phenotypes of pediatric bipolar disorder. Bipolar Disorders, 7(6). 10.1111/j.1399-5618.2005.00265.x 16403179

[jcv212060-bib-0038] Rich, B. , Brotman, M. , Dickstein, D. , Michell, D. , Blair, R. , & Leibenluft, E. (2010). Deficits in attention to emotional stimuli distinguish youth with severe mood dysregulation from youth with bipolar disorder. Journal of Abnormal Child Psychology, 38(5), 695–706. 10.1007/s10802-010-9395-0 20180010 PMC2880646

[jcv212060-bib-0039] Rich, B. , Carver, F. , Holroyd, T. , Rosen, H. , Mendoza, J. , Cornwell, B. , Fox, N. A. , Pine, D. S. , Coppola, R. , & Leibenluft, E. (2011). Different neural pathways to negative affect in youth with pediatric bipolar disorder and severe mood dysregulation. Journal of Psychiatry Research, 45(10), 1283–1294. 10.1016/j.jpsychires.2011.04.006 PMC3158808 21561628

[jcv212060-bib-0040] Rich, B. , Grimley, M. , Schmajuk, M. , Blair, K. , Blair, R. , & Leibenluft, E. (2008). Face emotion labeling deficits in children with bipolar disorder and severe mood dysregulation. Development and Psychopathology, 20(2), 529–546. 10.1017/S0954579408000266 18423093 PMC2669935

[jcv212060-bib-0041] Rich, B. , Schmajuk, M. , Perez‐Edgar, K. , Fox, N. , Pine, D. , & Leibenluft, E. (2007). Different psychophysiological and behavioral responses elicited by frustration in pediatric bipolar disorder and severe mood dysregulation. American Journal of Psychiatry, 164(2), 309–317. 10.1176/ajp.2007.164.2.309 17267795

[jcv212060-bib-0042] Savitz, D. A. , Wellenius, G. A. , & Trikalinos, T. A. (2019). The problem with mechanistic risk of bias assessments in evidence synthesis of observational studies and a practical alternaitve: Assessing the impact of specific sources of potential bias. American Journal of Epidemiology, 188(9), 1581–1585. 10.1093/aje/kwz131 31145434

[jcv212060-bib-0043] Shamseer, L. , Moher, D. , Clarke, M. , Ghersi, D. , Liberati, A. , Petticrew, M. , Shekelle, P. , Stewart, L. A. , & PRISMA‐P Group . (2015). Preferred reporting items for systematic review and meta‐analysis protocols (PRISMA‐P) 2015: Elaboration and explanation. Bmj, 349, g7647. 10.1136/bmj.g7647 25555855

[jcv212060-bib-0044] Steinberg, L. (2008). A social neuroscience perspective on adolescent risk‐taking. Developmental Review, 28, 78–106. 10.1016/j.dr.2007.08.002 18509515 PMC2396566

[jcv212060-bib-0045] Stoddard, J. , Hsu, D. , Reynolds, R. C. , Brotman, M. A. , Ernst, M. , Pine, D. S. , Leibenluft, E. , & Dickstein, D. P. (2015). Aberrant amygdala intrinsic functional connectivity distinguishes youths with bipolar disorder from those with severe mood dysregulation. Psychiatry Research: Neuroimaging, 231(2), 120–125. 10.1016/j.pscychresns.2014.11.006 PMC4370426 25544024

[jcv212060-bib-0046] Stoddard, J. , Sharif‐Askary, B. , Harkins, E. , Frank, H. , Brotman, M. , Penton‐Voak, I. , Moaz, K. , Bar‐Haim, Y. , Munafò, M. , Pine, D. S. , & Leibenluft, E. (2016). An open pilto study of training hostile interpretation bias to treat disruptive mood dysregulations disorder. Journal of Child and Adolescent Psychopharmacology, 26(1), 49–57. 10.1089/cap.2015.0100 26745832 PMC4779288

[jcv212060-bib-0047] Stoddard, J. , Stringaris, A. , Brotman, M. , Montville, D. , Pine, D. , & Leibenluft, E. (2014). Irritability in child and adolescent anxiety disorders. Depression and Anxiety, 31(7). 10.1002/da.22151 PMC3937265 23818321

[jcv212060-bib-0048] Stoddard, J. , Tseng, W. , Kim, P. , Chen, G. , Yi, J. , Donahue, L. , Brotman, M. , Towbin, K. , Pine, D. S. , & Leibenluft, E. (2017). Association of irritability and anxiety with the neural mechanisms of implicit face emotion processing in youths with psychopathology. JAMA Psychiatry, 74(1), 95. 10.1001/jamapsychiatry.2016.3282 27902832 PMC6309540

[jcv212060-bib-0049] Stringaris, A. , Goodman, R. , Ferdinando, S. , Razdan, V. , Muhrer, E. , Leibenluft, E. , & Brotman, M. (2012). The affective reactivity index: A concise irritability scale for clinical and research settings. Journal of Child Psychology and Psychiatry, 53(11), 1109–1117. 10.1111/j.1469-7610.2012.02561.x 22574736 PMC3484687

[jcv212060-bib-0050] Stringaris, A. , Ribas, P. , Brotman, M. A. , & Leibenluft, E. (2018). Vidal?Practitioner review: Definition, recognition, and treatment challenges of irritability in young people. Journal of Child Psychology and Psychiatry, 59(7), 721–739. 10.1111/jcpp.12823 29083031

[jcv212060-bib-0051] Sturm, A. , Rozenman, M. , Chang, S. , McGough, J. , McCracken , J. , & Piacentini, J. (2018). Are the components of social reciprocity transdiagnostic across pediatric neurodevelopmental disorders? Evidence for common and disorder‐specific social impairments. Psychiatry Research, 264, 119–123. 10.1016/j.psychres.2018.03.063 29627697 PMC6092937

[jcv212060-bib-0052] Teicher, M. H. , Andersen, S. L. , Polcari, A. , Anderson, C. M. , Navalta, C. P. , & Kim, D. M. (2003). The neurobiological consequences of early stress and childhood maltreatment. Neuroscience & Biobehavioral Reviews, 27(1–2), 33–44. 10.1016/S0149-7634(03)00007-1 12732221

[jcv212060-bib-0053] Thomas, L. , Brotman, M. , Bones, B. , Chen, G. , Rosen, B. , Pine, D. , & Leibenluft, E. (2014). Neural circuitry of masked emotional face processing in youth with bipolar disorder, severe mood dysregulation, and healhty volunteers. Developmental Cognitive Neuroscience, 8(C), 110–120. 10.1016/j.dcn.2013.09.007 24239048 PMC3960306

[jcv212060-bib-0054] Thomas, L. , Brotman, M. , Muhrer, E. , Rosen, B. , Bones, B. , Reynolds, R. , Deveney, C. M. , Pine, D. S. , & Leibenluft, E. (2012). Parametric modulation of neural activity by emotion in youth with bipolar disorder, youth with severe mood dysregulation, and healthy volunteers. Archives of General Psychiatry, 69(12), 1257. 10.1001/archgenpsychiatry.2012.913 23026912 PMC3538086

[jcv212060-bib-0055] Thomas, L. , Kim, P. , Bones, B. , Hinton, K. , Milch, H. , Reynolds, R. , Adleman, N. E. , Marsh, A. A. , Blair, R. J. R. , Pine, D. S. ,& Leibenluft, E. (2013). Elevated amygdala responses to emotional faces in youths with chronic irritability or bipolar disorder. NeuroImage: Clinical, 2, 637–645. 10.1016/j.nicl.2013.04.007 23977455 PMC3746996

[jcv212060-bib-0056] Tseng, W. , Deveney, C. , Stoddard, J. , Kircanski, K. , Frackman, A. , Yi, J. , Hsu, D. , Moroney, E. , Machlin, L. , Donahue, L. , Roule, A. , Perhamus, G. , Reynolds, R. , Roberson‐Nay, R. , Hettema, J. M. , Towbin, K. E. , Stringaris, A. , Pine, D. S. , Brotman, M. A. , & Leibenluft, E. (2019). Brain mechanisms of attention orienting following frustration: Associations with irritability and age in youths. American Journal of Psychiatry, 176(1), 67–76. 10.1176/appi.ajp.2018.18040491 30336704 PMC6408218

[jcv212060-bib-0057] Tseng, W.‐L. , Thomas, L. , Harkins, E. , Pine, D. , Leibenluft, E. , & Brotman, M. (2016). Neural correlates of masked and unmasked face. Social Cognitive and Affective Neuroscience, 11(1). 10.1093/scan/nsv087 PMC4692312 26137973

[jcv212060-bib-0058] Uran, P. , & Kilic, B. (2015). Comparison of neuropsychological performances and behavioral patterns of children with attention deficit hyperactivity disorder and severe mood dysregulation. European Child & Adolescent Psychiatry, 24(1). 10.1007/s00787-014-0529-8 24619769

[jcv212060-bib-0059] Uran, P. , & Kilic, B. (2020). Family functioning, comorbidities, and behavioral profiles of children with ADHD and disruptive mood dysregulation disorder. Journal of Attention Disorders, 24(9). 10.1177/1087054715588949 26078400

[jcv212060-bib-0060] Vidal‐Ribas, P. , Brotman, M. , Valdivieso, I. , Leibenluft, E. , & Stringaris, A. (2016). The status of irritability in psychiatry: A conceptual and quantitative review. Journal of the American Academy of Child & Adolescent Psychiatry, 55(7), 556–570. 10.1016/j.jaac.2016.04.014 27343883 PMC4927461

[jcv212060-bib-0061] Watson, D. , Stanton, K. , & Clark, L. (2017). Self‐report indicators of negative valence constructs within the research domain criteria (RDoC): A critical review. Journal of Affective Disorders, 216, 58–69. 10.1016/j.jad.2016.09.065 27823854

