# Supplementary material for: Underlying mechanisms of disruptive mood dysregulation disorder in children: A systematic review by means of research domain criteria
Source: JCPP Adv. 2022 Feb 18;2(1):e12060. doi: 10.1002/jcv2.12060 (PMC10242926; doi:10.1002/jcv2.12060)
Supplement: Supplementary file 1 — Supporting Information 1 [file JCV2-2-e12060-s001.docx]

**Appendix S1.**

**Supporting Information A.**

*Methods*

*Oppositional defiant disorder with chronic irritability and anger.* Originally, our search strategy was designed to include research on oppositional defiant disorder with irritability and anger (ODD-IA). ODD-IA was introduced by ICD-11 (WHO, 2018) as the equivalent to DSM-5’s DMDD. Thus, ODD-IA can be considered as a diagnostic category of clinic irritability. However, search terms aiming to identify research on ODD-IA yielded no results by September 2020. Thus, ODD-IA was excluded from the introduction and discussion of this systematic review, also due to space limitations.

**Supporting Information B.**

*Example of search terms initially explored in Ovid.*

| **Embase Classic+Embase, Ovid MEDLINE(R) ALL, APA PsycInfo** | | | | |
| --- | --- | --- | --- | --- |
| **DMDD (1)** | **Children (2)** | **Neurocognition (3)** | **Social perception (4)** | **Arousal (5)** |
| severe irritab* or chronic irritab* or persisten* irritab* or tonic irritab* or phasic irritab*  OR  ((Reactive aggressi* or temper outburst* or temper tantrum*) and irritab*)  OR  DMDD or (Disruptive and mood and dysregulation and disorder) or (disruptive and mood and dysregulation) or (severe and mood and dysregulation and disorder) or (severe and mood and dysregulation) | child* OR youth* OR pediatric* | neurocogniti* or executive function* or neuropsychology or cogniti* process* or exp cognitive processes or memory or inhibition or learn* or language or cogniti* or cogniti* system* or perception or cognitive control | exp Social processes or exp social perception or exp social cognition or theory of mind or social processes or social perception or social cognition or affect recognition or emotion recogniti* or exp emotion recognition or understanding of others or understanding of self | Stress or hydrocortisone or cortisol or hypothalamic pituitary adrenal axis or HPA or salvia or arousal or regulatory system* or circadian rythms |
| 2323 (1) | 6538010 (2) | 7342612 (3) | 759830 (4) | 2848016 (5) |
| 1420 (1 AND 2)  Remove duplicates: 835  English language: 792 | | 392 (1 AND 2 AND 3) | 46 (1 AND 2 AND 4) | 175 (1 AND 2 AND 5) |
| 525 (1 AND 2 AND (3 OR 4 OR 5))  *(Emba: 147, Medl: 66, Psyc: 105)* | | | | |
| Removed duplicates: 354 | | | | |
| English language: 346 | | | | |

**Supporting Information C.**

*Search terms optimized and translated to databases in Ovid.*

| **Database** | | **Search terms** | |
| --- | --- | --- | --- |
| Embase | | ((((Disruptive OR severe) ADJ mood dysregulation) OR (((persistent* OR chronic) ADJ3 irritab*) AND (temper ADJ3 (outburst* OR tantrum*)))) AND (child* OR youth* OR pediatric*)).tw. AND ((cogniti* OR social OR arousal OR threat OR reward OR motor).tw. OR exp cognition/ OR exp Social behavior/) | |
| MEDLINE | | ((((Disruptive or severe) ADJ mood dysregulation) OR (((persistent* OR chronic) ADJ3 irritab*) AND (temper ADJ3 (outburst* OR tantrum*)))) AND (child* OR youth* OR pediatric*)).tw. AND ((cogniti* OR social OR arousal OR threat OR reward OR motor).tw. OR exp Mental Processes/ OR exp Communication/ OR exp Psychophysiology/) | |
| PsycInfo | | ((((Disruptive or severe) ADJ mood dysregulation) OR (((persistent* OR chronic) ADJ3 irritab*) AND (temper ADJ3 (outburst* OR tantrum*)))) AND (child* OR youth* OR pediatric*)).tw. AND ((cogniti* OR social OR arousal OR threat OR reward OR motor).tw. OR exp cognitive processes/ OR exp social behavior/) | |
| *  ADJn  Exp … /  .tw. | : Truncation symbol  : Proximity  : Thesaurus term including narrower term(s)  : Words appears in the title or the abstract only | |  |

**Supporting information D.**

*Inclusion and exclusion criteria.*

|  | **Inclusion criteria** | **Exclusion criteria** |
| --- | --- | --- |
| **Period of published literature** | All | None |
| **Language** | English | Non-English |
| **Population** | 6-12, adjusted to 6-18 | <6-12<, adjusted to <6-18< |
| **Study design** | Quantitative: observational, experimental | Qualitative, review, meta-analysis, case study |
| **Publication type** | Peer reviewed journal article | Book, chapter, letter, review, note, conference abstract |
| **Indicator** | - DMDD (cf. DSM) - SMD (cf. Leibenluft et al., 2003) - ODD-IA (cf. ICD) | - Intellectual disability/mental retardation or somatic syndromes. - DMDD, SMD, or ODD-IA cannot be separated by other diagnosis by methods. - Diagnostic criteria not cf. DSM or ICD |
| **Comparison** | Children without DMDD, SMD or ODD-IA. | Children with DMDD, SMD or ODD-IA. |
| **Outcome** | RDoC units of analysis (genes, molecules, cells, circuits, physiology, behavior, subjective report, and paradigms) | Not in line with RDoC units of analysis (genes, molecules, cells, circuits, physiology, behavior, subjective report, and paradigms) |
| **Other** | Relevant cf. RDoC Domains (Social Processes, Cognitive Systems, Arousal/Regulatory Systems, Negative or Positive Valence Systems, Sensorimotor Systems). | - Full-text not available. - Results not related to RDoC domains (Social Processes, Cognitive Systems, Arousal/Regulatory Systems, Negative or Positive Valence Systems, Sensorimotor Systems). - Results not related to DMDD, SMD or ODD-IA |

**Supporting Information E.**

*Excluded Full Texts with reason*

| **Reference** | **Reason for exclusion** |
| --- | --- |
| Wiggins, Briggs-Gowan, Brotman, Leibenluft & Wakschlag (2021) | Indicator, Outcome (e.g., symptom level outcomes, i.e., no examination of underlying biological or cognitive contributors) |
| Uran & Kilic (2020) | Outcome (parent-report; WISC-R results not reported) |
| Chang, Chang, Cheng & Tzang (2020) | Comparison (DMDD could not be separated from ADHD) |
| Derella, Burke, Romano-Verthelyi, Butler & Johnston (2020) | Outcome (i.e., treatment) |
| Benarous, Renaud, Breton, Cohen, Réal & Guilé (2020a) | Outcome (data extracted from medical charts) |
| Benarous, Bury, Lahaye, Desroiers, Cohen & Guilé (2020b) | Outcome (parent-report) |
| Pan & Yeh (2019)* | Outcome (irritability, i.e., not related to any RDoC domain, and functional findings related to irritability and not DMDD). |
| Basu & Isaacs (2019) | Indicator, Outcome (childhood stressor, immigration) |
| Strum, Rozenman, Chang, McGough, McCracken & Piacentini (2018) | Outcome (parent-ratings) |
| Kunze, Wang, Isensee, Schlack, Ravens-Sieberer, Klasen, Rothenberger & Becker (2018) | Indicator (i.e., irritability measure) |
| Kessel, Dougherty, Kujawa, Hajcak, Carlson & Klein (2016) | Indicator (Age 3 DMDD symptoms, Age 9 no DMDD measure) |
| Salum, Mogg, Bradley, Stringaris. Gadelha, Pan, Rohde, Planczyk, Manfro, Pine & Leibenluft (2017)* | Indicator (i.e., irritability measure) |
| Perepletchikova, Nathanson, Axelrod, Merrill, Walker, Grossman, Rebeta, Scahill, Kaufman, Flye, Mauer & Walkup (2017) | Comparison (i.e., no comparison group without DMDD) |
| Dougherty, Barrios, Carlson & Klein (2017) | Comparison (no comparison group without DMDD) |
| Wiggins, Brotman, Adleman, Kim, Oakes, Reynolds, Chen, Pine & Leibenluft (2016) | Population (ages between 9-21 years) |
| Waxmonsky, Waschbusch, Belin, Li, Babocsai, Humphery, Parisea, Babinski, Hoffman, Haak, Mazzant, Fabiano, Pettit, Fallahazad & Pelham (2016) | Outcome (i.e., treatment) |
| Usami (2016) | Comparison (i.e., not possible to separate from ADHD) |
| Mitchell, Timmins, Collins, Scavone, Iskric & Goldstein (2016) | Comparison (DMDD could not be separated from bipolar disorder) |
| Mayes, Waxmonsky, Calhoun & Bixler (2016) | Indicator (i.e., symptom ratings, not full criteria DMDD), Outcome |
| Fristad, Wolfson, Guillermo, Algorta, Youngstrom, Arnold, Birmaher, Horwitz, Axelson, Kowatch, Findling & the LAMS Group (2016) | Outcome (symptom level, i.e., not related to underlying mechanisms) |
| Dougherty, Smith, Bufferd, Kessel, Carlson & Klein (2016) | Outcome (depressive or anxiety symptoms at age 9) |
| Stoddard, Stringaris, Brotman, Montville, Pine & Leibenluft (2014)* | Outcome (irritability) not relevant to any RDoC domain |
| Waxmonsky, Wymbs, Pariseau, Belin, Waschbusch, Babocsai, Fabiano, Akinnusi, Haak, & Pelham (2013) | Indicator, Comparison, Outcome (e.g., ADHD and SMD could not be separated, and no comparison group) |
| Copeland, Angold, Costello, & Egger (2013) | Outcome (estimated prevalence and comorbidity of DMDD) |
| Serra Giacobo, Jané, Bonillo, Ballespí, & Díaz-Regañon (2011) | Indicator, Outcome (e.g., SMD symptoms only, i.e., not diagnostic criteria). |
| Carlson, Potegal, Margulies, Basile & Gutkovich (2010) | Outcome (i.e., examine the safety and efficacy of liquid risperidone) |
| Carlson, Potegal, Margulies, Gutkovich, & Basile (2009) | Outcome (i.e., examined rage) |
| Dickstein, van der Veen, Knopf, Towbin, Pine & Leibenluft (2008) | Outcome (i.e., myo-inositol, glutamate/glutamine, *N*-acetyl aspartate relative to creatine during Tesla MRS in four brain region of interest) |

*Note.* *Unsure of decision.

**Supporting Information F.**

*Selecting approach for risk of bias assessment*

In the initial drafts of this systematic review, relevant questions from the Critical Appraisal Skills Programme (CASP) checklist for Case Control Studies (CASP, 2018) was conducted on the extracted papers to assess risk of bias. The Newcastle-Ottawa Scale (Wells et al., 2018) and Cochrane tools (Cochrane, 2021) were also considered as tools for addressing risk of bias, but the Cochrane tools is developed for intervention studies. Considering important comments from the JCPPA referees, however, following a practical guide by Savitz et al. (2019) was concluded as the most meaningful method for assessing risk of bias in the included studies of this review (i.e., non-randomized observational, etiology studies). To our knowledge, more mechanistic tools for assessing such studies is currently lacking.

Critical Appraisal Skills Programme (2018). Retrieved 12 21, 2020 from CASP Case Control Study Checklist: https://casp-uk.net/wp-content/uploads/2018/03/CASP-Case-Control-Study-Checklist-2018_fillable_form.pdf

Wells, G. A., Shea, B., Connell, D. O., et al. (2021, October 29) The Newcastle-Ottawa Scale (NOS) for assessing the quality of nonrandomised studies in meta analyses. <http://www.ohri.ca/programs/clinical_epidemiology/oxford.asp>.

Cochrane (2021, November 2). Cochrane Methods Bias, Resources. <https://methods.cochrane.org/bias/resources>

**Supporting Information G.**

*Risk of bias: Sample affiliation.*

The samples in twenty-three of the included studies were identified as affiliated with the U.S. NIMH (see Table 1). These studies stated that the participants enrolled in an ongoing NIMH study without further details, except for the studies published after 2016 where a study protocol identifier was provided demonstrating that six studies included participants from the same protocol (see table below). Four NIMH studies states that no SMD data was previously published (Adleman et al., 2012; Dickstein et al., 2010; Thomas et al., 2013; Rich et al., 2011).

|  | **Affiliation** | **Protocol** | **Protocol2** | **Protocol3** | **Comment** |
| --- | --- | --- | --- | --- | --- |
| Tseng et al. 2019 | NIMH, EDB | NCT00006177 | NCT00018057 | NCT00025935 | NIMH recruited 2012-2016 |
| Kircanski et al. 2018 | NIMH |  | NCT00018057 | NCT00025935 |  |
| Pagliaccio et al. 2017 | NIMH, EDB | NCT00006177 |  | NCT00025935 |  |
| Stoddard et al. 2017 | NIMH, EDB, SBSD | NCT00006177 | NCT00018057 | NCT00025935 |  |
| Freeman et al. 2016 | Other1 |  |  |  | Additional details of design and sample are available (Youngstrom et al. 2005); Recruited from all clinical intakes at a large community mental health center in the Midwestern US using a consecutive case series design |
| Stoddard et al. 2016 | NIMH, EDB, SBSD | NCT00006177 |  | NCT00025935 |  |
| Tseng et al. 2016 | NIMH, EDB |  |  | NCT00025935 |  |
| Uran & Kilic 2015 | Other2 |  |  |  | Children referred to Ankara University School of Medicine Child and Adolescent Psychiatry Department between May 2011 and 2012 |
| Perlman et al. 2015 | Other3 |  |  |  | recruited from local child psychiatric clinics (Clinical) |
| Hommer et al. 2014 | NIMH, EDB |  |  |  | were recruited into an IRB approved study at the National Institute of Mental Health |
| Thomas et al. 2014 | NIMH, EDB |  |  |  | Enrolled in an IRB-approved study at the NIMH |
| Thomas et al. 2013 | NIMH, EDB |  |  |  | Enrolled in an IRB approved protocol at the NIMH. No SMD data published previoysly, byt data from HV and BD published in Kim et al. 2012 |
| Kim et al. 2013 | NIMH, EDB |  |  |  | Enrolled in an IRB–approved protocol at the NIMH |
| Deveney et al. 2013 | NIMH, EDB |  |  |  | Enrolled in a study at the NIMH |
| Thomas et al. 2012 | NIMH, EDB |  |  |  | Enrolled in an IRB–approved protocol at the NIMH |
| Deveney et al. 2012a | NIMH, EDB |  |  |  | Enrolled in an IRB–approved protocol at the NIMH |
| Deveney et al. 2012b | NIMH, EDB |  |  |  | Enrolled in an IRB–approved study on BD at the NIMH |
| Adleman et al. 2012 | NIMH, EDB |  |  |  | Full recruitment and diagnostic methods are described previously (Brotman et al., 2010). but none of the data in the study have been published previously |
| Adleman et al. 2011 | NIMH |  |  |  | Recruitment described in Leibenluft et al. (2003) and Brotman et al. (2010) |
| Rich et al. 2011 | NIMH |  |  |  | Enrolled in an IRB-approved study at the NIMH.While BD and control data are published (Rich et al., 2010b), the data on SMD youth have not been presented previously. |
| Brotman et al. 2010 | NIMH, EDB |  |  |  | Enrolled in an IRB-approved study at the NIMH. Data from 20 bipolar disorder patients and 12 healthy comparison subjects have been published previously. Thus, among the 127 participants studied, data from 95 have not been presented previously |
| Dickstein et al. 2010 | NIMH, MAP |  |  |  | Enrolled in IRB-approved studies conducted at the NIMH Intramural Research Program |
| Rich et al. 2010 | NIMH, MAP |  |  |  | Enrolled in an IRB-approved study at the NIMH |
| Rau et al. 2008 | NIMH, MAP |  |  |  |  |
| Rich et al. 2008 | NIMH |  |  |  | Enrolled in an IRB-approved study at the NIMH |
| Guyer et al. 2007 | NIMH, MAP |  |  |  | Enrolled in studies at the NIMH |
| Dickstein et al. 2007 | NIMH |  |  |  | Were enrolled in two IRB-approved studies at NIMH |
| Rich et al. 2007 | NIMH, MAP |  |  |  | We enrolled subjects in an institutional review board-approved study at the NIMH |
| Rich et al. 2005 | NIMH |  |  |  | The NIMH IRB approved the two studies from which data were collected, an ongoing neurocognitive and neuroimaging study at the NIMH |

*Note.* EDB: Emotion and Development Branch, NIMH, National Institutes of Health, US Department of Health and Human Services, Bethesda, MD, US; MAP: Mood and Anxiety Disorders Program, NIMH, National Institutes of Health, Department of Health and Human Services (DHHS), Bethesda, MD, USA; NIMH: U.S. National Institute of Mental Health, Department of Health and Human Services; SBSD: Section on Bipolar Spectrum Disorders; Other1: Department of Psychology, University of Nevada, Las Vegas, Nevada; Department of Psychology, University of North Carolina at Chapel Hill, Chapel Hill, North Carolina; Bloomberg Children’s Center, Division of Child and Adolescent Psychiatry, Johns Hopkins University, Baltimore, Maryland; Other2: Child and Adolescent Psychiatry Department, Hatay Woman; Children Hospital, Antakya, Turkey; Other3: Department of Psychiatry, Western Psychiatric Institute and Clinic, University of Pittsburgh, United States b Department of Medical Social Sciences, Feinberg School of Medicine & Institute for Policy Research, Northwestern University, United States c Nationwide Children’s Hospital, United States, The Ohio State University School of Medicine, United States.
